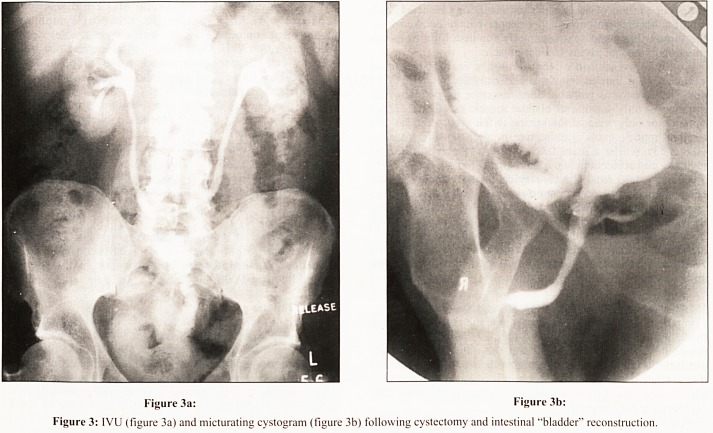# Current Trends in the Management of Invasive Bladder Cancer

**Published:** 1992-12

**Authors:** G. N. A. Sibley, J. Kabala

**Affiliations:** Departments of Urology and Radiology Bristol Royal Infirmary; Departments of Urology and Radiology Bristol Royal Infirmary


### Current Trends in the Management of Invasive Bladder Cancer

**G. N. A. Sibley and J. Kabala**
Departments of Urology and Radiology
Bristol Royal Infirmary

### INTRODUCTION

Bladder cancer is the fifth most common tumour in men and the tenth in women, with 8,000 new cases presenting annually in the UK. Seventy per cent of tumours are superficial at diagnosis (i.e. confined to the mucosa and submucosa, stages Ta and Tl) and can usually be managed endoscopically by resection and diathermy. Metastases are uncommon and the prognosis is generally good in this group of patients, although 10-15% will eventually progress to invasive disease.

In contrast, 30% of tumours have already invaded the underlying bladder muscle at presentation (stages T2-T4). Once muscle invasion has occurred, the prognosis is considerably poorer with a high risk of developing metastases (e.g. to lungs, liver and bone). A more radical approach to treatment should therefore be considered, and recent developments in management now give grounds for some optimism about improving the prognosis and quality of life in these patients. However, selection of patients for potentially curative radical treatment is dependent on accurate staging of the disease.

### CLINICAL ASSESSMENT OF TUMOUR STAGE

The local extent of the tumour is traditionally determined by cystoscopy and bimanual examination. At cystoscopy, the exophytic portion of the bladder tumour is first resected flush with the bladder wall and then a deeper biopsy is taken from the tumour base and sent separately for histology to assess invasion of the underlying bladder muscle.

Following resection of the tumour, a careful bimanual examination is carried out. In superficial tumours (stages Ta and Tl), no mass is palpable after resection. In patients with invasion of only superficial bladder muscle (stage T2), either no mass or only residual thickening is felt. The presence of a residual mobile mass is indicative of deep muscle invasion (stage T3), whilst a mass fixed within the pelvis indicates invasion into adjacent pelvic tissues (stage T4).

Unfortunately, local clinical staging has an accuracy of only 80% with regard to the primary tumour. In addition, it does not provide any information with regard to lymph node or distant metastases, both of which are crucial in determining which patients may be candidates for radical curative treatment. However, modern imaging techniques now provide much valuable information that is helping to improve staging accuracy.

### IMAGING TECHNIQUES FOR STAGING

Computed tomography (CT) has become the standard imaging modality for staging bladder cancer over the last decade, proving a significant advance over the methods previously available.[1] However, it is clear that CT is unable to reliably differentiate the stage of tumours confined to the bladder or to identify perivesical involvement, and prediction of lymph node involvement may be poor.[2]

Magnetic resonance imaging (MRI) has recently emerged as a powerful tool for the staging of bladder cancer (Fig. l). There is some variability in the published data, but an accuracy of 85-95% has been suggested for the prediction of extravesical spread.[3]

Initial data suggested that MRI, like CT, could not differentiate between different stages of bladder wall invasion. This has to a certain extent been challenged, with at least one series reporting the ability to differentiate between superficial and deep muscle invasion with an accuracy of 94%.[4]

**Figure 1:** Coronal T1W scan following Gadolinium injection. The urine shows a high signal. Multiple tumour nodules are shown projecting into the bladder lumen (intermediate signal) but they are clearly confined to the bladder and do not demonstrate any extravesical extension. The two nodules seen over the dome of the bladder have also been shown to be superficial, separate from the deep muscle of the bladder wall in contrast to the tumour mass at the bladder base which is likely to be invading into the deep muscle layer.

Using an upper limit of normal of 1 cm diameter, the sensitivity of MRI for the detection of lymph node involvement is around 50-83%, with a (remarkable) reported specificity of up to 100%.[3,4] These figures suggest that MRI is reliably detecting secondaries when the lymph nodes are enlarged but, like CT, is unable to diagnose lymph nodes containing micrometastases.

MRI continues to develop, and thus further increases in its accuracy for staging bladder tumours is likely. The use of a double surface coil has already been reported to increase spatial resolution and clinical accuracy. Intravenous enhancement with Gadolinium may afford better differentiation of tumour stage within the bladder wall and aid in the assessment of lymph node involvement.[5]

### MANAGEMENT

In the absence of nodal or distant metastases, T2 and T3 tumours are potentially curable. Treatment may involve radiotherapy, chemotherapy or surgery, or combinations of each of these modalities.

### (a) T2 tumours

In patients in whom there is no residual mass palpable in the bladder wall after tumour resection, it is reasonable to assume that the muscle biopsy has removed all the viable tumour. In this situation a well-differentiated tumour can be followed up as if it were a superficial one. If there is thickening palpable after resection or if the tumour is poorly-differentiated, then a more radical approach to treatment is indicated, as for T3 tumours.

### (b) T3 tumours

In deeply invasive bladder cancer, the disease has progressed beyond endoscopic control and radical treatment is indicated.

External beam radiotherapy (40-60 Gy) has been the generally preferred treatment in the UK for muscle-invasive tumours, and remains the treatment of choice in many centres.[6]

**Figure 2:** Schematic representation of intestinal "bladder" reconstruction. (Courtesy of Medical Illustration and the Medical Artist, Gary James).

**Figure 3a:**

**Figure 3b:**

**Figure 3:** IVU (figure 3a) and micturating cystogram (figure 3b) following cystectomy and intestinal "bladder" reconstruction.

After radiotherapy, regular endoscopic follow-up remains essential. If viable tumour is still present in the bladder following radiotherapy or if invasive tumour subsequently recurs, total cystectomy and urinary diversion is indicated (**salvage cystectomy**). Cystectomy may also be required for severe side effects after radiotherapy (bleeding, contracted bladder, incontinence, fistulae). If, however, tumour recurrences after radiotherapy are superficial, they can be managed by repeated cystodiathermy without resort to radical surgery.

In the USA and in some centres in Britain, there is a trend towards aggressive initial surgical treatment of muscle-invasive bladder cancer by total cystectomy, which classically involves removal of the lower ureters, bladder, prostate and urethra in men, and the lower ureters, bladder, urethra and gynaecological organs in women. Although the results of cystectomy (either alone or following pre-operative radiotherapy) are superior to radiotherapy alone,[7] surgery has been less popular in the past because of the need for simultaneous urinary diversion (by ileal conduit or uretero-sigmoidostomy).

However, with recent developments in reconstructive surgical techniques, it is now possible in suitable cases to avoid urinary diversion by construction of a "bladder substitute" from the intestine that is then anastomosed directly to the membranous urethra. The intestinal "bladder" may be constructed from ileum[8] (Fig. 2), the sigmoid colon or using an ileo-caecal segment. This provides a continent reservoir that is emptied by abdominal straining (Fig.3), although intermittent self-catheterisation may be required to achieve complete emptying. This technique is applicable mainly for male patients, in whom potency may also be retained by careful preservation of the neurovascular bundles innervating the corpora cavernosa. However, it is contraindicated in patients with multifocal tumours or widespread carcinoma in situ due to the risks of urethral recurrence.

When anastomosis to the membranous urethra is not feasible (in women and in all patients where the urethra must be removed), the bowel reservoir can be given a continent catheterisable stoma instead. The continence mechanism can be provided by submucosal tunnelling of a narrow tube, such as appendix or ureter, into the reservoir (Mitrofanoff principle), or by intussusception of a length of ileum to form a non-return valve (the Kock pouch).[9] The patient then empties the reservoir at regular intervals by self-catheterisation of the cutaneous stoma opening onto the abdominal wall.

The role of radical surgery in the presence of lymph node metastases is controversial. Although radical pelvic node dissection may be curative in patients with only microscopic evidence of disease, the overall survival is probably not improved in patients with bulky nodal metastases.

Whilst radiotherapy and surgery are effective in achieving local control of invasive bladder tumours, only 40-50% of patients survive 5 years and a significant proportion eventually succumb to metastatic disease that was undetected at presentation (micro-metastases). This has led to interest in the use of systemic chemotherapy in conjunction with radical local treatment to try and improve survival, and clinical trials are currently in progress to evaluate this. Regimes include a combination of cisplatin, methotrexate and vinblastine (CMV)[10], and a multicentre trial of this regime given in a "neo-adjuvant" fashion prior to radical local treatment is currently being conducted by the Medical Research Council. In other studies, adriamycin is also incorporated into the regime (M-VAC).

### (c) T4 tumours

The prognosis for these tumours is very poor, and treatment is palliative. Radiotherapy may relieve local symptoms such as haematuria, and palliative chemotherapy may be helpful in carefully selected cases. The terminal event is often renal failure due to ureteric obstruction.

### (d) Metastatic bladder cancer

With the development of chemotherapy regimes active against invasive bladder cancer, reasonable palliation can be obtained in selected patients with metastases. A 30% response rate can be achieved in terms of a measurable decrease in the size of tumour deposits, although significant toxicity may be incurred. Chemotherapy seems to be most effective for nodal and pulmonary metastases, and is relatively ineffective for hepatic or bone secondaries.

### SUMMARY

In muscle-invasive bladder cancer, attempts at cure have traditionally involved radical local treatment by either radiotherapy or ablative surgery. However, these treatments have been associated with a high morbidity and have failed to address the problem of subsequent metastatic disease, to which many patients eventually succumb (often within the first 3 years after treatment).

Modern imaging techniques have led to much improved staging information, allowing careful selection of patients suitable for radical "curative" treatment; at the same time, patients identified as already having metastatic disease may be spared major surgery that is unlikely to influence the outcome of their disease.

Reconstructive surgical techniques are beginning to transform the quality of life for patients offered radical surgery, by avoiding the need for traditional urinary diversion. In addition, the use of neo-adjuvant chemotherapy combined with radical local treatment addresses the problem of micro-metastases at diagnosis and offers the prospect of improved survival, although the results of clinical trials are awaited to evaluate this further.

Future advances in treatment may be expected to occur as our understanding of the biology of bladder cancer increases. Of particular value will be predictive information about the invasive potential of initially superficial tumours, so that these cases may be targeted for "aggressive" treatment from the outset.

### REFERENCES

1. Sager E. M., Talle K., Fossa S., Ous S., Stenwig A. E. (1983). The role of CT in demonstrating perivesical growth in the preoperative staging of carcinoma of the urinary bladder. *Radiology*: **146**: 443-446.
2. Vosges G. E., Tauschke E., Stockle M., Alken P., Hohenfellner R. (1989). Computerized tomography. An unreliable method for accurate staging of bladder tumours in patients who are candidates for radical cystectomy. *J.Urol.*: **142**: 972-974.
3. Buy J. N, Moss A., Guinet C., Ghossain M. A., Malbec L., Arrive L., Vadrot D. (1988). MR staging of bladder carcinoma: correlation with pathologic findings. *Radiology*: **169**: 695-700.
4. Tavares N.J., Demas B.E., Hricak H. (1990). MR imaging of bladder neoplasms: correlation with pathologic staging. *Urol.Radiol.*: **12**: 27-33.
5. Neuerburg J. M., Bohndorf K., Sohn M., Teufl F., Guenther R. W., Daus H. J. (1989), Urinary bladder neoplasms: evaluation with contrast-enhanced MR imaging. *Radiology.*: **172**: 739-743.
6. Jenkins B. J., Caulfield M. J., Fowler C. G., Badenoch D. F., Tiptaft R. C., Paris A. M. I., Hope-Stone H. F., Oliver R. T. D., Blandy J. P. (1988). Reappraisal of the role of radical radiotherapy and salvage cystectomy in the treatment of invasive (T2/T3) bladder cancer. *Br.J.Urol.*: **62**: 343-346.
7. Sternberg C. N., Scher H. I. (1989). Management of invasive bladder neoplasms. In: *Smith P. H. (ed) Combination Therapy in Urological Malignancy. Springer-Verlag, Berlin.*: pp 95-118.
8. Studer U. E., Ackermann D., Casanova G. A., Zingg E. J. (1989). Three years experience with an ileal low pressure bladder substitute. *Br.J.Urol.*: **63**: 43-52.
9. Skinner D. G., Lieskovsky G., Boyd S. (1989). Continent urinary diversion. *J.Urol.*: **141**: 1323-1327.
10. Fossa S. D., Harland S. J., Kaye S. B., Raghavan D., Russell J. M., Parmar M. K. B., Uscinska B. M., Wood R. (1992). Initial combination chemotherapy with cisplatin, methotrexate and vinblastine in locally advanced transitional cell carcinoma - response rate and pitfalls. *Br.J.Urol.*: **70**: 161-168.

## Figures and Tables

**Figure 1 f1:**
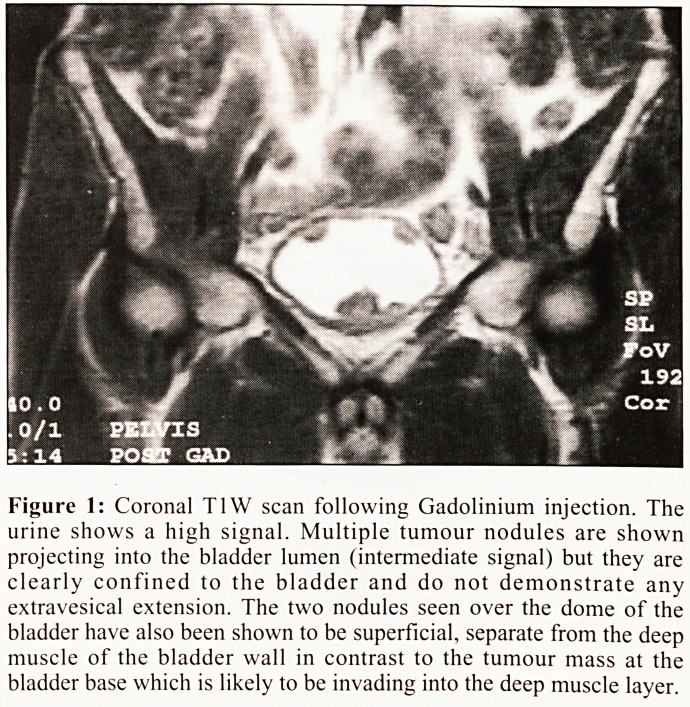


**Figure 2 f2:**
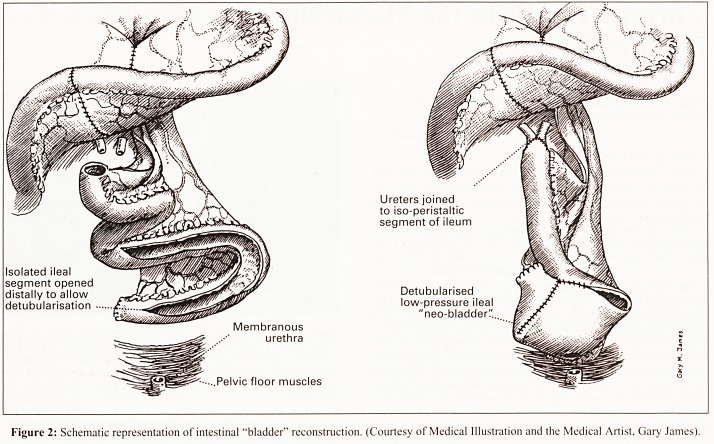


**Figure 3a Figure 3b f3:**